# Rhythmic Foot Embrocation According to Wegman/Hauschka for Alleviating Symptoms of Chemotherapy-Induced Peripheral Neuropathy: A Randomized Controlled Trial

**DOI:** 10.1177/15347354251382931

**Published:** 2025-11-12

**Authors:** Burcu Babadağ Savaş, Judith Büntzel, Sandra Liebscher-Koch, Bettina Märtens, Heike Meyer, Annette Sander, Maren Schürmann, Axel Weiser, Tatjana Zielke, Yvonne Ziert, Diana Steinmann

**Affiliations:** 1 Department of Radiotherapy, Hannover Medical School, Hannover, Germany; 2 Department of Hematology and Medical Oncology, University Medical Center Göttingen, Göttingen, Germany; 3 Nursing Science Working Group/Complementary Nursing, University Medical Center Göttingen, Göttingen, Germany; 4 Klaus-Bahlsen-Center for Integrative Oncology, Comprehensive Cancer Center Lower Saxony, Hannover Medical School, Hannover, Germany; 5 Pediatric Hematology and Oncology, Hannover Medical School, Hannover, Germany; 6 Quality Management, Hannover Medical School, Hannover, Germany; 7 Institute of Biostatistics, Hannover Medical School, Hannover, Germany

**Keywords:** rhythmic embrocation, care, chemotherapy-induced peripheral neuropathy, cancer, nursing

## Abstract

**Background:**

Chemotherapy-induced peripheral neuropathy (CIPN) in patients treated with platinum-, taxane- or vinca alkaloid-based chemotherapy may cause symptoms such as tingling, numbness, pain, and paresthesia, particularly in patients’ hands and feet. This study aimed to evaluate rhythmic embrocation (RE) of the feet according to Wegman/Hauschka in addition to an exercise program (EP) as a complementary treatment for CIPN symptoms.

**Methods:**

This study was a prospective, randomized controlled, 2-center trial. A total of 57 patients with CIPN symptoms were randomly allocated into 2 groups, with 52 patients analyzed: the intervention group (n = 26) and the control group (n = 26). While the intervention group received 3 RE according to Wegman/Hauska and EP within 14 days, the control group performed only EP. CIPN symptoms (*tingling, numbness, pain and cramps*) were evaluated with the Numeric Rating Scale (NRS), and quality of life related to peripheral neuropathy was assessed with the EORTC QLQ-CIPN20 questionnaire.

**Results:**

The mean NRS scores for total CIPN symptoms (tingling, numbness, pain, and cramps) decreased from baseline to 24 hours after the third intervention in both groups. However, the intervention group maintained lower scores 2 weeks later, whereas the scores in the control group returned to baseline levels. A significant time effect was observed for NRS scores with a medium effect size (*P* < .001, η² = 0.122), but no significant difference was found between the groups (*P* > .05). Similarly, significant time effects were observed in the *sensory and motor subgroups* of the EORTC QLQ-CIPN20 (*P* < .001), although the between-group differences remained nonsignificant (*P* > .05). While the intervention group showed greater improvements, particularly at 2 weeks post-intervention, a statistically significant difference between the groups was not reached.

**Conclusion:**

According to our study results, RE combined with exercise, particularly after 3 interventions, was more effective in reducing CIPN symptoms in the short-term than was exercise alone; however, symptoms increased again in the absence of interventions (standard care) by the end of the fourth week.

## Introduction

The incidence of chemotherapy-induced peripheral neuropathy (CIPN) in patients treated with platinum-, taxane- or vinca alkaloid-based chemotherapy ranges from moderate to high.^1^ CIPN causes symptoms such as tingling, numbness, pain, and paresthesia, particularly in patients’ hands and feet.^2^ These symptoms can affect the quality of life, general functional health and daily activities of affected patients.^2^ Studies have shown that CIPN is associated with pain, fatigue, anxiety, depression, psychological distress, sleep problems and decreases physical mobility.^3-5^ Due to the lack of effective management of patients’ symptoms, individuals may experience losses in mobility, independence in daily tasks, and roles related to family, home, and work. This is particularly evident in CIPN, where the absence of a pharmacological treatment capable of fully curing or eliminating symptoms leads to recurrent patient complaints.^5,6^ As a result, nonpharmacological approaches are also utilized to address these challenges. For instance, the updated 2025 version of the German S3 Guideline “Supportive therapy for oncology patients” recommends the implementation of regular functional training as a potential prophylactic intervention.^7^ In addition, for taxane-associated CIPN, both cryotherapy and compression therapy are endorsed as effective preventative measures. The guideline further supports the inclusion of exercise and physical training as part of the therapeutic regimen for patients experiencing established CIPN symptoms.^7^ Both American Society of Clinical Oncology (ASCO)^8^ and European Society for Medical Oncology (ESMO)^9^ guidelines do not strongly recommend exercise, acupuncture or scrambler therapy for CIPN due to limited and inconsistent evidence. Additionally, many nursing applications, such as aromatherapy and/or massage applications on the hands and feet of patients, especially during oncological treatment, have been reported in the literature to reduce neuropathic symptoms.^4,6^ In the field of manipulative and body-based practices, rhythmic embrocation (RE) is a promising method developed by the physician Ita Wegman in the 1920s.^10,11^ The physician Dr Margarethe Hauschka continued to work on the application of RE, resulting in a professional and teachable concept that became widespread in clinics and home care from the 1980s onward.^11,12^ The application of rhythmic massage has positive effects on reducing pain and symptoms of illness as well as on the quality of life of chronically ill patients.^10,13^ RE relaxes muscles; supports vital processes such as breathing, peristalsis and sleep; stimulates warmth in the extremities; and alleviates functional complaints such as pain and tiredness.^12^ Foot embrocation has calming, grounding, and relaxing effects and promotes sleep. RE is a supportive treatment whose health-promoting effect is aimed primarily at perception and self-healing processes^14^ Oils or ointments are used to support the soothing effect.^8^ It is assumed that the symptoms of polyneuropathy, such as pain, tingling and numbness in the feet, which develop during or after chemotherapy treatment due to neurotoxic substances, can be reduced by RE according to Wegman/Hauschka. In particular, gentle circular and linear RE are practised in clinical settings by nurses trained in this technique in some clinics in German-speaking countries.^15,16^ However, to date, no experimental study has investigated the effect of Wegman/Hauschka rhythmic embrocation on CIPN complaints in the feet.

Therefore, the objective of this study was to evaluate RE of the feet according to Wegman/Hauschka, applied with limited frequency, in addition to an exercise program (EP) as a complementary treatment to reduce CIPN symptoms in the short-term.

## Materials and Methods

### Study Design

This study was a prospective, randomized, controlled, 2-center trial conducted in the oncology departments of 2 tertiary hospitals (Department of Radiotherapy, Hannover Medical School (MHH) and Clinic for Hematology and Medical Oncology, University Medical Center Göttingen (UMG)) as a part of the Comprehensive Cancer Center in Lower Saxony (CCC-N). At the MHH, the treatments were carried out as part of the outpatient integrative oncology service (Klaus-Bahlsen-Zentrum); at the UMG, they were carried out as part of the inpatient oncology service by complementary nursing staff.

### Study Populations

The study sample included oncology patients who consented to participate and met the following criteria: (a) aged 18 to 95 years; (b) diagnosed with an oncological disease and presenting symptoms of CIPN; (c) had received at least 1 course of platinum, taxane, or vinca alkaloid-based chemotherapy or therapy involving proteasome inhibitors, thalidomide, lenalidomide, or pomalidomide; (d) reported a score of ≥4/10 on the Numeric Rating Scale (NRS) for pain, cramps, tingling, or numbness in the feet^3^; (e) had no coagulopathies and maintained a platelet count of >100 000/µL^3,4^; (f) exhibited no infections or open wounds on the feet; and (g) had no other neuropathies, such as diabetic polyneuropathy or related conditions (h) had no allergies to the base oil (almond oil).

### Allocation to Study Arms and Sample Size Calculation

**Figure 1.**
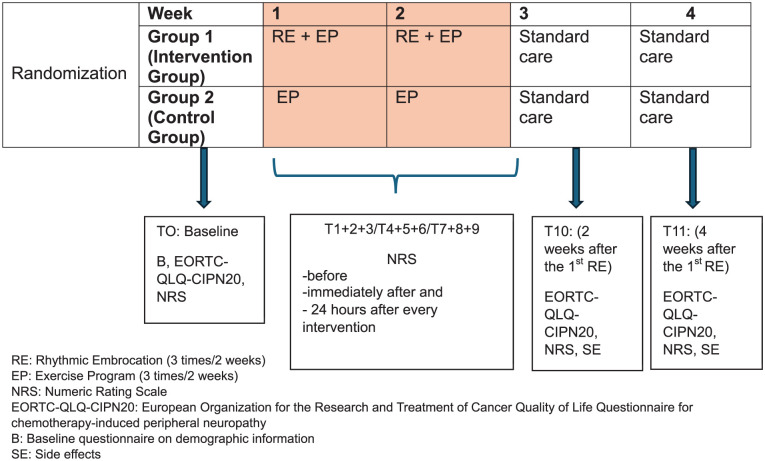
Study design. Abbreviations: RE, rhythmic embrocation (3 times/2 weeks); EP, exercise program (3 times/2 weeks); NRS, numeric rating scale; EORTC-QLQ-CIPN20, European Organization for the Research and Treatment of Cancer Quality of Life Questionnaire for chemotherapy-induced peripheral neuropathy; B, baseline questionnaire on demographic information; SE, side effects.

Patients who met the inclusion criteria were randomized to intervention group (Group 1; RE + exercise program (EP)) or control group (Group 2; EP; Figure 1). We used a randomization procedure with an allocation ratio of 1:1. The subjects were randomly assigned to the 2 groups using a computerized randomizer.^17^

**Figure 2.**
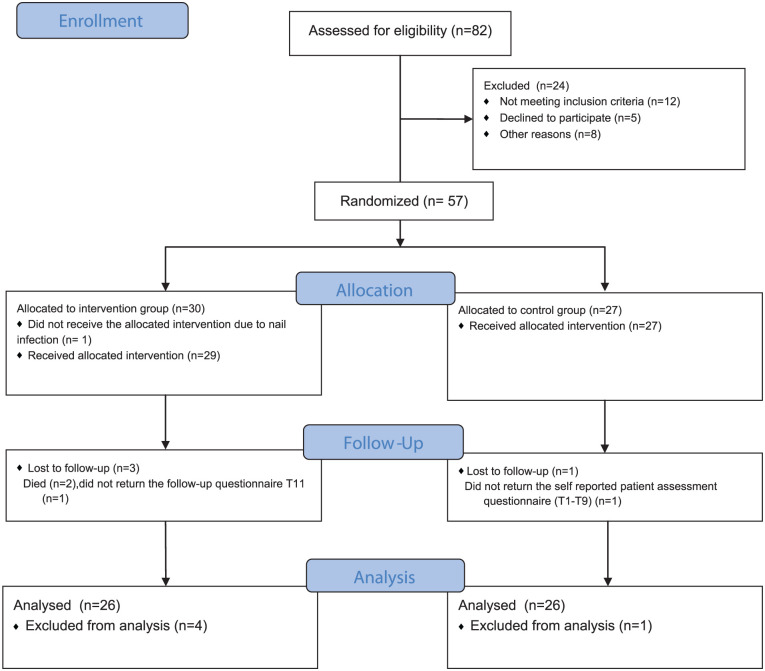
Patient flow diagram (consort 2010 flow diagram).

The sample size was powered to detect a mean difference of 2.08 with a standard deviation of 2.3 between the groups with 80% power at a significance level of 5% in the primary outcome. Therefore, 48 (intervention group n = 24 and control group n = 24) patients were included. Concerning possible drop-outs, we recruited a sample of N = 57 patients, and 52 patients (intervention group, n = 26; control group, n = 26) were analyzed. Figure 2 shows the patient flowchart. The sample size calculation was performed using nQuery 9.

### Intervention

**Figure 3.**
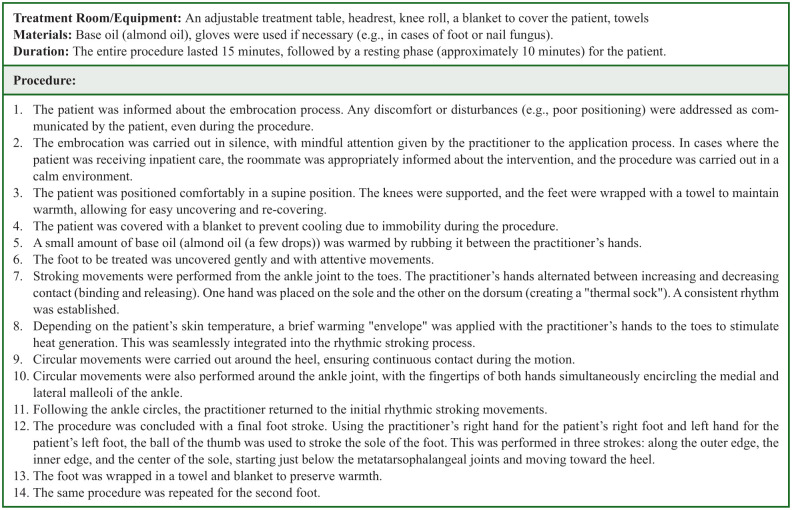
RE according to Wegman/Hauschka procedure.

**Figure 4.**
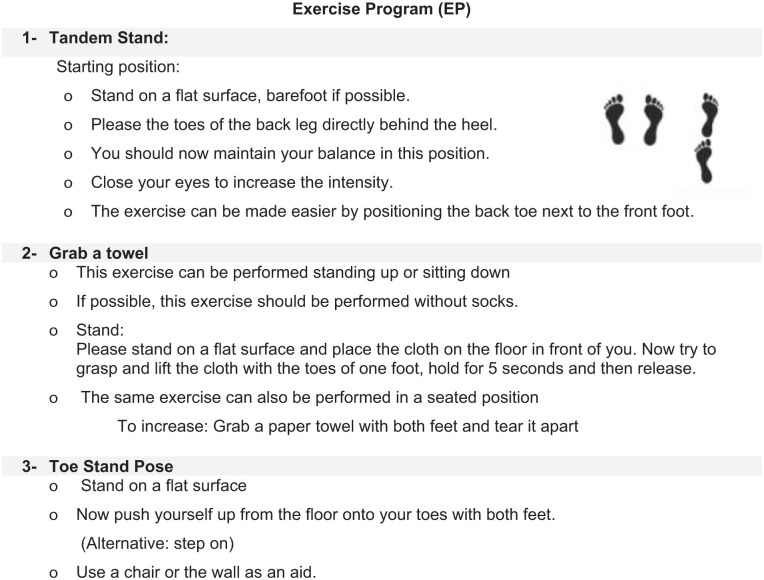
Exercise program.

*Intervention group (Group 1)*: RE was performed on the patients according to Wegman/Hauschka, with application of base oil (almond oil) on the feet, total of 3 times over a 14-day period, with a minimum interval of 2 days between each session. For example, although many sessions were conducted on days 5, 10, and 14, the exact timing varied slightly among participants depending on clinical feasibility, but the minimum 2-day interval rule was maintained. RE on the feet was carried out for 15 minutes by nurses who had received special training in RE. This procedure is shown in Figure 3. The patients in the intervention group also carried out an exercise program 3 times over the 14 days (the details of which are described below and in Figure 4).

*Control group (Group 2)*: In the S3 guideline “Supportive therapy for oncology patients,” regular exercise training is recommended as part of the nonmedication therapy for patients with CIPN.^17,18^ Therefore, an exercise program was developed by the Department of Sport Medicine, MHH, according to the S3 guideline recommendation “Complementary medicine in the treatment of oncological patients” for implementation at home.^19^ Patients therefore performed the EP at least 3 times over a period of 14 days. The interval between interventions was a minimum of 2 days. Patients who had been randomized to the control group were offered RE after completion of the last study follow-up.

Basic exercise principles were demonstrated using illustrated materials to both the control group (routine care) and the intervention group to ensure clarity and standardization (Figure 4).

### Measurement

The data were collected using the questionnaires described below, via the online platform “SoSci Survey” or through a paper-and-pencil format.

### Form to Collect Sociodemographic Data and Disease and Treatment Characteristics

This form was developed according to related literature^4,6,20^ and consists of items related to sociodemographic data and disease and treatment characteristics.

### Numeric Rating Scale (NRS)

The NRS is an instrument that assesses patients’ tolerance of painful paresthesia. It is a Likert-type scale ranging from 0 to 10, with 0 representing no pain and 10 representing the worst pain.^4^ Each CIPN symptom (*pain, tingling, cramps, and numbness*) was rated on a Numeric Rating Scale (ranging from 0 to 10) and the sum of these scores was used to determine the overall CIPN symptom score (ranging from 0 to 40).

### European Organization for the Research and Treatment of Cancer Quality of Life Questionnaire for Chemotherapy-Induced Peripheral Neuropathy (EORTC QLQ-CIPN20)

The EORTC QLQ-CIPN20 is a patient self-report questionnaire developed by Postma et al^21^ to document symptoms and functional limitations associated with CIPN in patients exposed to potentially neurotoxic chemotherapeutic and/or neuroprotective agents and is available in multiple validated translations, including German.^21,22^ It is a 4-point Likert-type scale *ranging from 1 (not at all) to 4 (very much)* consisting of 20 items. The scale has 3 subscales, namely, sensory, *motor and autonomic.* The CIPN20 scale includes questions related to symptoms such as *pain, tingling, cramps, or numbness* in the feet. All subscores, as well as the questions specifically addressing these 4 CIPN symptoms in the feet, were calculated according to the formula of the EORTC QLQ-C30 symptom scale.^21^ For all subscale scores, higher values indicate increased severity of symptoms and functional limitations.^6,21^

### Questionnaire for Assessing Side Effects

This form, consisting of 2 items, was prepared by the researchers to determine whether the patient experienced any side effects during or after the intervention. If side effects occurred, the type and duration were to be specified.

The questionnaire was administered 2 and 4 weeks after the first intervention, and participants were asked to report any side effects that occurred during or within the 2 weeks following the intervention sessions. Thus, the first evaluation covered the period from week 0 to week 2, and the second from week 2 to week 4.

## Outcomes

### Primary Outcome

The primary endpoint was change in pain levels in relation to CIPN symptoms measured via the NRS; scores recorded before the first application of RE were compared with those recorded 24 hours after the third treatment.

### Secondary Outcome

The secondary endpoints included NRS scores recorded 24 hours, 2 weeks, and 4 weeks after the first RE, as well as an assessment of polyneuropathy symptoms using the EORTC QLQ-CIPN20 questionnaire at 2 and 4 weeks after the initiation of RE.

### Assessment

An overview of the study design is presented in Figure 1. Following the collection of patient information and consent (T0 = baseline; RE/EP), sociodemographic data, disease and treatment characteristics, NRS scores (pain, cramps, tingling, numbness) and quality of life related to polyneuropathy (EORTC QLQ-CIPN20) data were collected using the appropriate data collection instruments. Before, immediately after and 24 hours after each application (T1 + 2 + 3/T4 + 5 + 6/T7 + 8 + 9), patients indicated their complaints according to the NRS. The nurses who administered the intervention reminded the patients to perform the evaluations immediately before and after the application. At the end of the intervention phase (after 2 weeks; T10) and 4 weeks after the first RE or EP (T11), the patients were asked again about NRS (pain, tingling, numbness, and cramps), quality of life with polyneuropathy (EORTC QLQ-CIPN20) and side effects of the RE/EP (questionnaire for assessing side effects).

### Statistical Analyses

Statistical analyses were performed using IBM SPSS Statistics (Statistical Package for Social Sciences Version 29.0.1.1). The normal distribution of metric data was evaluated using the Kolmogorov-Smirnov test and a histogram. Descriptive analyses (means, standard deviations, absolute and relative frequencies) were used to describe the data. A total of 5 patients were excluded from the analysis due to insufficient data. Specifically, in the intervention group: death due to poor prognosis (n = 2) nail infection (n = 1) and lack of participation in the evaluation (n = 1); and in the control group: lack of participation in the evaluation (n = 1). A Per-Protocol (PP) analysis was conducted, as only participants who completed the intervention and provided sufficient outcome data were included in the final analysis (Figure 2).

Pearson’s chi-square test, Student’s *t* test and the Mann-Whitney-*U* test were employed to examine the differences in sociodemographic, disease, and treatment characteristics between the 2 groups. Repeated measures of CIPN symptoms, assessed using the NRS and the EORTC QLQ-CIPN20 scales, were analyzed and compared between groups using 1-way repeated-measures ANOVA. Eta-squared (η^2^) effect sizes were calculated to express the mean score differences of groups between the measurement times. Effect sizes were defined as follows: η^2^ ≥ 0.01, small effect; η^2^ ≥ 0.06, medium effect; and η^2^ ≥ 0.14, large effect.^23^ Statistical significance was set at *P* < .05. SPSS, by default, conducts analyses by dropping cases for which there are missing values, so the sample sizes may differ in the statistical analyses. Inferential statistics are used in a descriptive manner. Thus, neither global nor local significance levels were determined, and no adjustment for multiplicity was applied.

### Ethical Considerations

The research project was approved by the ethics committees of MHH and UMG (approval number: 10291_BO_S_2022) and registered in the German Clinical Trials Register (DRKS) with the ID DRKS00029743. Furthermore, all participants provided informed consent and acknowledgment of the data privacy policy before being included in the study. The study was conducted in accordance with the CONSORT guidelines.^24^

## Results

### Sociodemographic Data and Disease and Treatment Characteristics of the Study Participants

The mean age of the patients was 61.54 ± 10.07 years; 75.0% were female, 42.3% had breast cancer and had 88.8% undergone treatment with a curative intent. The mean time since the initiation of CIPN symptoms (*tingling, numbness, pain or cramps*) in the feet was 29.12 ± 37.03 months in the intervention group and 37.12 ± 73.21 months in the control group. A total of 66.2% of patients in the intervention group and 46.2% in the control group had completed cancer treatment. In the intervention group, 53.8% of patients received taxane-based agents, 34.7% platinum-based agents, 7.7% proteasome inhibitors, and 3.8% lenalidomide. In the control group, 46.2% received platinum-based agents, 34.6% taxane-based agents, 11.5% proteasome inhibitors, and 7.7% vinca alkaloids. The mean time since the completion of chemotherapy was 16.83 ± 19.79 months in the intervention group and 63.42 ± 103.74 months in the control group. A total of 57.7% of patients in the intervention group and 69.2% of the patients in the control group did not currently use pain medication for CIPN symptoms. Both groups were similar in terms of sociodemographic data and disease and treatment characteristics, such as age, sex, diagnosis, duration of symptoms, severity of symptoms, and treatment (*P* > .05; Table 1). All participants in the intervention group completed the 3 scheduled RE sessions, and the majority of them (96.0%, n = 25) received treatment as outpatients.

**Table 1.** Sociodemographic-, Disease-, and Treatment Characteristics of Participants (N = 52).

Characteristic	Sample size		Intervention group		Control group		Statistical analysis
	n	Mean ± SD	Median (min-max)	n	Mean ± SD	Median (min-max)	*P*
Age (y)	26	58.92 ± 10.08	59 (32-75)	26	63.15 ± 9.55	65 (48-80)	.060^a^
Time since CIPN symptoms (months)	25	29.12 ± 37.03	9 (2-132)	24	37.12 ± 73.21	12 (1-360)	.952^b^
NRS (T0 - baseline)*	26	17.23 ± 9.17	15 (6-38)	26	15.84 ± 7.97	13 (5-34)	.819^b^
EORTC QOL CIPN 20 (T0 - Baseline)							
Sensory	26	48.57 ± 14.22	46 (22-89)	26	50.14 ± 20.53	50 (15-96)	.750^a^
Motor	26	34.93 ± 16.66	38 (4-79)	26	36.05 ± 22.07	38 (0-75)	.837^a^
Autonomic	26	5.55 ± 20.66	0 (−11-55)	26	12.82 ± 21.24	6 (11-66)	.128^b^
	n	%		n	%		*P*
Gender							
Female	21	80.8		18	69.2		.337^c^
Male	5	19.2		8	30.8		
Diagnose							
Breast cancer	15	57.7		7	26.9		.250^c^
Colorectal cancer	4	15.4		6	23.1		
Multiple Myeloma	2	7.7		3	11.5		
Ovarian cancer	1	3.8		3	11.5		
Lymphoma	0	0.0		2	7.7		
Others**	4	15.4		5	19.2		
Current cancer treatment							
Yes	8	30.8		14	53.8		.092^c^
No	18	69.2		12	46.2		
	n	Mean ± SD	Median (Min-Max)	n	Mean ± SD	Median (Min-Max)	
Time since completion of chemotherapy (months)	18	16.83 ± 19.79	11 (1-72)	12	63.42 ± 103.74	12 (3-360)	.299^b^
Chemotherapy treatment	n	%		n	%		*P*
Platinum	9	34.7		18	46.2		
Taxane	14	53.8		9	34.6		.336^c^
Vinca alkaloid	0	0.0		2	7.7		
Proteasome inhibitors	2	7.7		3	11.5		
Lenalidomide	1	3.8		0	0.0		
Current use of pain medications for CIPN symptoms							
Yes	11	42.3		8	30.8		.388^c^
No	15	57.7		18	69.2		
Current pain medication for CIPN symptoms							
NSAI (Ibuprofen, Paracetamol)	6	23.1		3	11.5		.736^c^
Gabapentin	4	15.4		4	15.4		
Opioid	1	3.8		1	3.8		

a Student’s t-test.

b Mann-Whitney *U* test.

c Pearson Chi-square test.

* Sum of the baseline CIPN symptoms (numbness, tingling, cramp, pain score) on numeric rating scale (0-40).

** Others (pancreatic cancer, ovarian cancer, neuroendocrine tumor, skin cancer etc.).

### NRS Score Comparisons on CIPN Symptoms

The mean NRS scores for total CIPN symptoms (tingling, numbness, pain, and cramps) tended to decrease from baseline (start of the first intervention) to 24 hours after the third intervention in both groups (intervention group from 13.85 ± 7.58 to 10.38 ± 6.89 vs the control group from 14.23 ± 7.67 to 12.08 ± 6.44). However, the mean NRS scores in the intervention group remained lower 2 weeks after the third intervention (from 13.85 ± 7.58 to 11.69 ± 7.62), whereas in the control group, they increased beyond the initial baseline (from 14.23 ± 7.67 to 14.76 ± 7.52). Additionally, during the 2 weeks between T10 and T11, when no intervention was applied, a slight increase was observed in the intervention group (from 11.69 ± 7.62 to 12.61 ± 8.26); however, the scores remained below the initial mean NRS scores. In the control group, although a slight decrease in scores was observed at the end of the 2 weeks without any intervention (from 14.76 ± 7.52 to 14.34 ± 6.71), this decrease remained above the initial baseline level.

The results revealed a significant and medium-sized time effect on the mean NRS scores in both groups (*P* < .001, η²* = 0.122*). However, the difference between groups was not significant, and the time × group interaction was also not significant (*P* > .05), indicating that the changes over time were similar for both groups (Table 2).

**Table 2.** Comparison of the CIPN Symptoms’ Mean Values Between Groups on the NRS.

NRS**	Measurement time	Intervention group (n = 26), Mean ± SD	Control group, (n = 26), Mean ± SD
	T1 (Start of first intervention)	13.85 ± 7.58	14.23 ± 7.67
	T9 (24 h after third intervention)	10.38 ± 6.89	12.08 ± 6.44
	T10 (2 wk after first intervention)	11.69 ± 7.62	14.76 ± 7.52
	T11 (4 wk after first intervention)	12.61 ± 8.26	14.34 ± 6.71
*P* values*	Time	*P* < .001 η^2^ = 0.122	
	Between group	*P* = .363 η^2^ = 0.017	
	Time × group	*P* = .248 η^2^ = 0.027	
	Before each intervention^a^	12.25 ± 6.40	13.00 ± 5.60
	Immediately after the intervention^b^	9.77 ± 5.64	12.86 ± 7.30
	24 h after the intervention^c^	10.70 ± 6.36	12.49 ± 6.12
*P* values*	Time	*P* < .001 η^2^ = 0.148	
	Between group	*P* = .279 η^2^ = 0.023	
	Time × group	*P* = .006 η^2^ = 0.112	

Abbreviations: NRS, numeric rating scale; n, number of patients; M, mean; SD, standard deviation.

a Sum of CIPN symptoms before each intervention (T1 + T4 + T9).

b Sum of CIPN symptoms immediately after the intervention (T2 + T5 + T8).

c Sum of CIPN symptoms 24 hours after the intervention (T3 + T6 + T9).

* One-way repeated measure ANOVA.

** Sum of the CIPN symptoms (tingling, numbness, pain, cramps score) on Numeric Rating Scale (0-40).

In addition, the most noticeable reduction in the mean NRS scores for total CIPN symptoms (tingling, numbness, pain, and cramps) was observed immediately after the intervention, with a slight tendency for an increase 24 hours later. The results revealed a significant and large time effect on the mean NRS scores in both groups (*P* < .001, η² = *0.148*). Although the intervention group showed a greater reduction (from 12.25 ± 6.40 to 9.77 ± 6.64) than the control group did (from 13.00 ± 5.60 to 12.86 ± 7.30), no significant difference was found between the groups (*P* > .05). However, the time × group interaction was significant, indicating that the time-dependent changes in the intervention group were significantly different from those observed in the control group, with a medium effect size (*P* = .006, η² = *0.112; Table 2*).

### EORTC QLQ CIPN20 Scale Score Comparison on CIPN Symptoms

A significant and large time effect was observed in the *sensory* (*P* < .001, η² = *0.281*) and *motor* (*P* < .001, η² = *0.212*) subgroups of the mean EORTC QLQ-CIPN20 scores within the groups. Although greater changes were observed in the intervention group than in the control group, particularly at 2 weeks after the first intervention, the difference between the groups was not statistically significant (*P* > .05). Additionally, the time × group interaction was not significant (*P* > .05), indicating that changes over time were similar for both groups. In terms of the *autonomic* subgroup of the EORTC QLQ-CIPN20, the mean scores were not significantly different between the groups (*P* > .05; Table 3).

**Table 3.** Comparison of the mean values of the groups EORTC QLQ CIPN20 scale.

EORTC QOL-CIPN 20 subgroups	Measurement time	Intervention group (n = 26), mean ± SD	Control group (n = 26), mean ± SD
Sensory	Baseline T0	48.57 ± 14.22	50.14 ± 20.53
	T10 (2 wk after first treatment)	39.17 ± 16.36	46.15 ± 21.75
	T11 (4 wk after first treatment)	39.03 ± 16.83	42.16 ± 20.17
P values	Time	*P* < .001 η^2^ = 0.281	
	Between group	*P* = .428 η^2^ = 0.013	
	Time × group	*P* = .172 η^2^ = 0.035	
Motor	Baseline T0	34.93 ± 16.66	36.05 ± 22.07
	T10 (2 wk after first treatment)	27.40 ± 16.25	31.41 ± 21.28
	T11 (4 wk after first treatment)	27.40 ± 19.08	28.04 ± 19.42
*P* values*	Time	*P* < .001 η^2^ = 0.212	
	Between group	*P* = .703 η^2^ = 0.003	
	Time × group	*P* = .515 η^2^ = 0.013	
Autonomic	Baseline T0	5.55 ± 20.66	12.82 ± 21.24
	T10 (2 wk after first treatment)	8.11 ± 20.50	11.53 ± 18.71
	T11 (4 wk after first treatment)	8.97 ± 23.09	7.69 ± 17.43
*P* values*	Time	*P* = .727 η^2^ = 0.006	
	Between group	*P* = .549 η^2^ = 0.007	
	Time × group	*P* = .089 η^2^ = 0.048	

Abbreviations: EORTC QLQ CIPN 20, European Organization for the Research and Treatment of Cancer Quality of Life Questionnaire for Chemotherapy-Induced Peripheral Neuropathy; n, number of patients; M, mean; SD, standard deviation.

* One way repeated measures ANOVA.

### NRS and EORTC QLQ CIPN20 Scale Score Comparison on Tingling, Numbness, Pain, Cramps

When comparing each individual CIPN symptom (*tingling, numbness, pain*, and *cramps*) in both groups, significant medium-sized reductions in mean NRS scores, particularly in numbness (*P* = .022, η^2^ = *0.076*) and *pain* (*P* = .019, η^2^ = *0.078*), were observed, especially 2 weeks after the first intervention. Although no significant differences were observed between groups, a significant difference was found in the time × group interaction, with the intervention group showing more pronounced changes and having a medium effect size (*numbness* from 5.96 ± 2.75 to 4.23 ± 2.82, *P* = .025, η^2^ *=* *0.073; pain* from 4.30 ± 3.45 to 2.35 ± 2.88, *P* = .018, η^2^ = *0.079*). With respect to the EORTC QLQ-CIPN20 scale, although no significant changes in *numbness* were observed between the groups (*P* > .05), a significant and large time difference in *pain* score (*P* < .001, η² = *0.160*) was found between the 2 groups. However, no significant differences in *pain* score were observed between the groups (*P* > .05; Table 4).

**Table 4.** Comparison of CIPN Symptoms (Tingling, Numbness, Pain, Cramps) Mean Values of Intervention and Control Group in NRS and EORTC QLQ CIPN20 Scale.

		NRS*		EORTC QLQ-CIPN 20**	
		Intervention group (n = 26)	Control group (n = 26)	Intervention group (n = 26)	Control group (n = 26)
CIPN Symptoms	Measurement time	Mean ± SD	Mean ± SD	Mean ± SD	Mean ± SD
Tingling	Baseline T0	4.73 ± 2.93	5.53 ± 2.68	74.36 ± 31.70	78.20 ± 29.73
	T10 (2 wk after first treatment)	3.92 ± 2.60	4.65 ± 2.75	60.26 ± 31.30	65.38 ± 33.30
	T11 (4 wk after first treatment)	3.65 ± 3.00	4.57 ± 2.61	55.13 ± 29.72	65.38 ± 35.88
*P* values***	Time	*P* < .001 η^2^ = 0.151		*P* < .001 η2 = 0.214	
	Between group	*P* = .252 η^2^ = 0.026		*P* = .428 η^2^ = 0.013	
	Time × Group	*P* = .928 η^2^ = 0.001		*P* = .580 η^2^ = 0.010	
Numbness	Baseline T0	5.96 ± 2.75	5.61 ± 2.43	75.64 ± 30.63	80.77 ± 25.25
	T10 (2 wk after first treatment)	4.23 ± 2.82	5.46 ± 2.74	64.10 ± 36.42	78.20 ± 22.98
	T11 (4 wk after first treatment)	4.46 ± 2.67	5.80 ± 2.62	65.38 ± 29.02	75.64 ± 24.14
*P* values***	Time	*P* = .022 η^2^ = 0.076		*P* = .052 η^2^ = 0.058	
	Between group	*P* = .245 η^2^ = 0.027		*P* = .155 η^2^ = 0.040	
	Time × group	*P* = .025 η^2^ = 0.073		*P* = .427 η^2^ = 0.017	
Pain	Baseline T0	4.30 ± 3.45	2.53 ± 3.11	62.82 ± 34.41	51.28 ± 37.98
	T10 (2 wk after first treatment)	2.35 ± 2.88	2.61 ± 3.09	44.87 ± 36.44	44.87 ± 35.20
	T11 (4 wk after first treatment)	2.80 ± 2.93	2.38 ± 2.80	42.30 ± 35.97	35.90 ± 33.89
*P* values***	Time	*P* = .019 η^2^ = 0.078		*P* < .001 η^2^ = 0.160	
	Between group	*P* = .392 η^2^ = 0.015		*P* = .491 η^2^ = 0.010	
	Time × group	*P* = .018 η^2^ = 0.079		*P* = .391 η^2^ = 0.019	
Cramps	Baseline T0	2.23 ± 2.73	2.15 ± 2.89	44.87 ± 36.44	39.74 ± 40.02
	T10 (2 wk after first treatment)	1.19 ± 2.09	2.03 ± 2.73	21.79 ± 32.58	34.61 ± 38.27
	T11 (4 wk after first treatment)	1.69 ± 2.69	1.58 ± 2.55	32.05 ± 31.94	30.77 ± 32.55
*P* values***	Time	*P* = .120 η^2^ = 0.042		*P* = .006 η^2^ = 0.100	
	Between group	*P* = .732 η^2^ = 0.002		*P* = .800 η^2^ = 0.001	
	Time × group	*P* = .226 η^2^ = 0.029		*P* = .113 η^2^ = 0.043	

Abbreviations: NRS, numeric rating scale; EORTC QLQ CIPN 20, European Organization for the Research and Treatment of Cancer Quality of Life Questionnaire for Chemotherapy-Induced Peripheral Neuropathy; n, number of patients; M, mean; SD, standard deviation.

* CIPN symptoms (numbness, tingling, cramp, pain score) on Numeric Rating Scale (0-10).

** Questions regarding numbness, tingling, cramp, or pain score on feet on EORTC QLQ CIPN20 Scale.

*** One-way repeated measures ANOVA.

In terms of mean *tingling* scores, significant time reductions were observed in both groups according to both the NRS (*P* < .001, η² = *0.151*) and EORTC QLQ-CIPN20 scale (*P* < .001, η² = *0.214*). However, the time × group interaction was not significant (*P* > .05), indicating that the changes over time were similar for both groups (Table 4).

With respect to *cramps* scores, although no significant differences were found according to the NRS (*P* > .05), there was a significant and medium-sized difference in both groups according to the EORTC QLQ-CIPN20 scale (*P* = .006, η² = *0.100*). Additionally, no significant differences were found in the time × group interaction for cramps, indicating similar changes over time between the groups (*P* > .05; Table 4).

### Evaluation of the Side Effects

No serious adverse events occurred in either group. At the 2-week follow-up, 7.7% of patients in the intervention group reported experiencing mild, short-term pain, burning, or tingling immediately after rhythmic embrocation. In comparison, 3.8% of patients in the control group experienced mild and short-term tingling or pain directly after performing the exercise program (*P* > .05). At the 4-week follow-up, no other side effects were reported in either group.

## Discussion

This study investigated the effects of RE of the feet according to Wegman/Hauschka in addition to EP as a complementary treatment for CIPN symptoms (*tingling, numbness, pain or cramps)*. According to the results of our study, within 2 weeks—particularly when evaluated before the first application and 24 hours after the last application—a slight decrease in CIPN symptoms was observed in the intervention group (RE and EP) compared with those in the control group that only performed exercises. However, these changes did not differ significantly between the groups, although both showed significant improvements over time. Furthermore, after the interventions were completed, no applications, only standard care, were performed for 2 weeks. By the end of this intervention-free period, symptom severity in the control group had increased beyond baseline levels, whereas in the intervention group, although symptoms also increased slightly, the progression was slower and remained below the initial symptom levels. To the best of our knowledge, this is the first randomized controlled study in the literature specifically addressing the reduction of CIPN symptoms through RE with an EP. Moreover, RE, according to Wegman/Hauschka, is a complementary method used by trained nurses in clinical practice to relieve pain, particularly in some individual clinics in German-speaking countries.^15,16^

A systematic review and an expert consensus process on the prevention and treatment of CIPN with nonpharmacological interventions, along with a consensus process conducted by a team of integrative oncology experts from German-speaking countries, discussed the potential benefits of complementary methods and clinical recommendations. Although there are no studies on rhythmic embrocation according to the results of a systematic review, clinical improvement after using RE (with aconite oil or arnica comp/formica oil) for the treatment of CIPN symptoms has been reported by consensus, with an efficacy rating of 4 on a scale of 0 to 5 on the basis of expert opinion.^15^ In addition, various complementary nursing practices—such as classical massage, aromatherapy massage, and rhythmic massage—have been used as manipulative therapies to prevent or treat CIPN symptoms in the literature.^4,6,15^

In agreement with the results of our study, Izgu et al reported that patients suffering from peripheral neuropathy who received 12 sessions of classical massage therapy as prevention experienced less peripheral neuropathic pain than did the control group. Additionally, at the end of 16 weeks, the control group showed an increase in pain from baseline levels.^6^ Therefore, as observed in our study, RE may affect peripheral neuropathy, with symptoms taking longer to return to the same severity in the RE group than in the control group. Moreover, in another study by Izgu et al, patients who received hand and foot aromatherapy massage as a treatment for CIPN symptoms 3 times a week reported fewer complaints of peripheral paresthesia than did patients in the control group at the end of the sixth week.^4^ In a study by Sarısoy and Ovayolu, in patients with non-Hodgkin lymphoma, classical foot massage administered 3 times per week for 4 weeks had a positive effect on the treatment of peripheral neuropathic pain and CIPN symptoms.^25^ A systematic review of external applications in integrative medicine by Mühlenpfordt et al reported that RE has physical effects, such as a warming sensation, improved sensitivity in the legs, and reduced pain and muscle relaxation (local), as well as psychological effects, such as feelings of release, liberation and relaxation. It was also reported that RE may have long-term effects and that these effects increase after multiple applications.^14^ Ostermann et al reported that repeated RE with solum oil in patients with chronic low back pain effectively reduced pain intensity and improved their ability to cope with pain.^10^ In addition, 1 of the other results of our study was that, compared to that in the control group, the most noticeable reduction in mean NRS scores for total CIPN symptoms was observed immediately after the application of RE, with a slight tendency for an increase 24 h later. Furthermore, the majority of patients in our study had been chronic sufferers of CIPN for a minimum of 1 year, making the observed positive outcomes after just 3 sessions of RE combined with EP particularly promising. Increasing the frequency of RE or continuing treatment with chemotherapy after the completion of chemotherapy may lead to more effective and long-term positive effects.

In addition to pain, CIPN also causes symptoms such as *tingling, numbness, and cramps*, as well as difficulties in *autonomic, sensory, and motor functions*.^26^ We also assessed CIPN symptoms (*tingling, numbness, pain, cramps)* individually. One of the findings of our study is that although improvements in specific *numbness* and in *pain*, *sensory and motor functions* were slightly greater in the group receiving RE combined with exercise, no significant differences were found between the 2 groups. In the literature, studies comparing manipulative therapies such as classical massage with a control group receiving no intervention have reported improvements in sensory, motor, and other CIPN symptom.^4,6^ Although greater improvements in CIPN symptoms were observed in the group receiving both RE and exercise, the lack of a control group receiving only standard care, the inability to assess manipulative interventions separately, and the small sample size may have contributed to the absence of significant differences between the groups.

CIPN is a complex condition due to the diversity of symptoms, the difficulty in defining them, their long-term persistence, and the lack of standardized medical treatment.^26^ Therefore, managing such complex symptoms requires a multidisciplinary team—including nurses, doctors, physiotherapists, nutritionists, occupational therapists, etc.—along with supportive multimodal interventions and medical treatment. In our study, RE was used alongside exercise. Similarly, in the exploratory phase 2 study by Schönsteiner et al , patients were evaluated using whole-body vibration training as an adjunct to an integrated program that included massage, passive mobilization, and physical exercise. Their findings suggested that a program incorporating massage, mobilization, physical exercise, and whole-body vibration had significant and clinically relevant benefits in alleviating CIPN symptoms, improving physical fitness, and enhancing sensory function. Moreover, such programs were recommended to be integrated into daily clinical practice and systematically assessed by nurses specializing in CIPN.^27^ Similarly, in the study by Wen and Cai, the effectiveness of preventing CIPN symptoms during oxaliplatin treatment for 1 to 6 cycles of chemotherapy was examined by comparing a control group receiving only conventional nursing care before chemotherapy with an experimental group receiving an integrated nursing model based on evidence-based interventions. In the experimental group, an expert team consisting of a psychologist, dietitian, clinical pharmacologist, physician, and nurse provided the integrated nursing model. Accordingly, in addition to the standard care received by the control group, patients in the experimental group were provided with functional exercises, emotional support, nutritional and health-related recommendations and traditional Chinese medicine (TCM)-based nursing applications such as application of a warm compressed wrapped in a sea salt or 50% magnesium sulfate, along the intravenously centered wet compress at the injection site or Golden powder mixed with honey. The study results indicated that the integrated nursing model during oxaliplatin treatment for 1 to 6 cycles of chemotherapy contributed to a reduced incidence of peripheral nerve injury and an improvement in quality of life.^28^ Therefore, as evidenced by our study findings, evidence-based multimodal nursing interventions, which incorporate multiple supportive approaches, may be more effective in managing complex symptoms such as CIPN.

## Limitations

This study has several limitations. Although the sample size was relatively small, it was determined based on a priori power analysis as described in the Methods section. However, the small sample size still limits the generalizability of the findings. There was no control group that received only classical massage and/or standard treatment for comparison with the RE group. Additionally, the intervention group did not include frequent RE or exercise applications; therefore, only short-term effects were assessed. Since the EP was performed by the patients at home, the level of adherence and whether they performed the exercises adequately were not assessed. Because side effects were only assessed retrospectively and focused on symptoms occurring during or after intervention, the findings may be prone to recall bias. Furthermore, the study sample consisted of a heterogeneous group of patients who experienced long-term CIPN symptoms and were undergoing treatment, and no sample group was included for preventive interventions. Due to the heterogeneity of the chemotherapy agents used, the effectiveness of the intervention could not be demonstrated in relation to any single drug. Additionally, chemotherapy toxicity was not assessed using standard evaluation tools such as the Chemotherapy Toxicity Criteria. Consequently, potential differences in patient responses regarding toxicity levels could not be taken into account in the analysis. Therefore, the use of standard toxicity assessment tools is recommended in future studies.

## Conclusion

According to our study results, RE combined with exercise, particularly after 3 interventions, was more effective in reducing CIPN symptoms in the short-term than was exercise alone; however, symptoms increased again in the absence of interventions (standard care) by the end of the fourth week. It is recommended that the intervention be continued beyond the 2-week period to maximize patient benefits. Additionally, short-term positive effects were observed in the intervention group, particularly in relation to sensory and motor functions, as well as numbness and pain associated with CIPN. Although the sample size in this study was determined through a priori power analysis, future studies are recommended to include larger sample sizes to better demonstrate the effectiveness of the intervention, allow for subgroup analyses, and improve generalizability. Additionally, more frequent repetitions, and in combination with other integrative multimodal approaches-particularly when performed by nurses trained in RE- may improve the overall effectiveness and clinical relevance of the intervention.

## Supplemental Material

sj-doc-1-ict-10.1177_15347354251382931 – Supplemental material for Rhythmic Foot Embrocation According to Wegman/Hauschka for Alleviating Symptoms of Chemotherapy-Induced Peripheral Neuropathy: A Randomized Controlled Trial Supplemental material, sj-doc-1-ict-10.1177_15347354251382931 for Rhythmic Foot Embrocation According to Wegman/Hauschka for Alleviating Symptoms of Chemotherapy-Induced Peripheral Neuropathy: A Randomized Controlled Trial by Burcu Babadağ Savaş, Judith Büntzel, Sandra Liebscher-Koch, Bettina Märtens, Heike Meyer, Annette Sander, Maren Schürmann, Axel Weiser, Tatjana Zielke, Yvonne Ziert and Diana Steinmann in Integrative Cancer Therapies
